# Dimensionally constrained adversarial attack and defense in wind power forecasting

**DOI:** 10.1371/journal.pone.0345284

**Published:** 2026-03-27

**Authors:** Yangming Min, Congmei Jiang, Liangheng Zhang, Xiankui Wen, Junling Tu, Jing Zhang

**Affiliations:** 1 College of electrical engineering, Guizhou University, GuiYang, Guizhou, China; 2 Guizhou University Survey and Design Institute Co., Ltd., Guiyang, Guizhou, China; 3 Electric Power Science Research Institute, Guizhou Power Grid Co., Ltd., Guiyang, Guizhou, China; 4 Zunyi Power Supply Bureau, Guizhou Power Grid Co., Ltd., Zunyi, Guizhou, China; Maulana Abul Kalam Azad University of Technology West Bengal, INDIA

## Abstract

Deep neural networks (DNNs) have achieved remarkable success in wind power forecasting, but DNNs are vulnerable to adversarial attacks that can severely degrade forecast accuracy. Existing studies primarily emphasize attack effectiveness and pay limited attention to attack stealthiness. In this paper, a dimension-constrained momentum iterative fast gradient sign method (DC-MI-FGSM) is proposed for wind power forecasting, which generates highly stealthy perturbations by applying the momentum update mechanism during attack optimization and limiting the perturbation dimensions of input samples. To defend against this attack, a denoising autoencoder (DAE)-based preprocessing defense strategy is developed for wind power forecasting, which resists adversarial attacks by mapping adversarial samples back to their corresponding clean forms. The effectiveness of the proposed attack and defense methods is validated on the public SDWPF dataset under both white-box and black-box scenarios. Compared with existing attacks, DC-MI-FGSM achieves a lower average perturbation percentage (APP), indicating superior attack stealthiness. Meanwhile, it causes more severe degradation in forecasting accuracy, as measured by MAPE, RMSE, and MAE, demonstrating stronger attack effectiveness. For defense, the proposed DAE-based preprocessing strategy effectively mitigates adversarial perturbations, significantly reducing forecasting errors while preserving the original accuracy on clean data. Moreover, it consistently outperforms adversarial training in terms of robustness and usability.

## Introduction

Conventional power generation relies on fossil fuels such as oil, coal and natural gas, which emit large quantities of harmful gases and seriously pollute the ecological environment [1]. In recent years, wind energy, as a renewable and environmentally friendly resource, has been used for power generation on a large scale [2,3]. However, wind power generation is affected by factors such as wind speed, wind direction, and temperature, leading to significant variability and uncertainty [4,5]. Therefore, accurate wind power forecasting is essential for power management and planning, effectively enhancing the economic and social benefits of power systems [6,7].

To improve wind power forecast accuracy, various machine learning methods have been employed, such as support vector machine regression (SVR) [8], random forest [9], autoregressive integrated moving average (ARIMA) [10], vector autoregression (VAR) [11], artificial neural networks (ANNs) [12]. However, the traditional machine learning techniques are relatively simple and rely heavily on manually crafted features, which might not achieve an ideal forecasting accuracy for complex and nonlinear wind power data. In recent years, deep neural networks (DNNs) [13–15] have been widely used to automatically capture the correlation between input samples and wind power output, often providing highly accurate forecast results [16]. Notable architectures include Long Short-Term Memory (LSTM) [17], Convolutional Neural Networks (CNNs) [16], Gated Recurrent Unit (GRU) [18], Temporal Convolutional Network (TCN) [19], and Transformer Neural Network [20]. These architectures can automatically capture long-term temporal dependencies within wind power data and spatial correlations across multiple wind farms, thereby more accurately fitting the mapping from the input samples to the wind power output.

Although DNN-based forecasting models have achieved high accuracy, their nonlinear nature also makes them vulnerable to adversarial attacks [21–23]. Input data such as wind speed, wind direction, and ambient temperature are critical to accurate forecasts and are typically obtained via online weather forecast application programming interfaces (APIs) [22]. During transmission over communication networks, such data may be intercepted or tampered with, exposing potential attack surfaces for adversarial manipulation [23]. If the input data used for wind power forecasting are compromised by such attacks, the resulting perturbations can lead to degradation in forecasting accuracy, which may have real-world consequences, including inaccurate dispatching, increased reserve requirements, and reduced economic benefits for wind farm operators. In [24], a universal adversarial perturbation attack is proposed for wind power forecasting, which generates a single offline perturbation to degrade forecasting errors across different models. Experimental results show that this method achieves attack effectiveness comparable to customized attacks while exhibiting stronger robustness. In [25], the projected gradient descent (PGD) is used to perform untargeted, semi-targeted, and targeted attacks on wind power data, thereby affecting the forecast results of the LSTM and CNN models to varying degrees in the white-box environment. In [26], an attack strategy targeting external-factor data of wind power is proposed and shown to be effective across multiple DNN-based forecasting models. This approach employs an attack sample selection model to improve stealthiness by selectively filtering the attack samples, and an attack direction judgment model to enhance the attack effectiveness by determining the correct attack direction. In [27], a new attack algorithm, called the adversarial learning attack, is proposed for wind power forecasting. This algorithm stably optimizes the meteorological data into its adversarial patterns, effectively degrading the forecast accuracy of the LSTM model in the white-box and black-box environments. Although existing studies have demonstrated the vulnerability of wind power forecasting models to adversarial attacks, most research primarily focuses on attack effectiveness, while little attention has been paid to the stealthiness of adversarial perturbations.

To ensure the safe application of DNNs, it is crucial to investigate the defense algorithms. Adversarial attacks in smart grids are usually characterized by complex data features, diverse attack strategies, and strong temporal correlations, which means that traditional defenses may not be directly effective for the power data [28,29]. In [30], a data replacement method for wind power forecasting is proposed to defend against adversarial attacks in the white-box environment. This method mitigates attacks by identifying perturbations in input samples and replacing corrupted samples with corresponding forecasted values. In [25], the effectiveness of the adversarial training (AT) [31] is demonstrated in wind power forecasting. This algorithm is a common defense algorithm that improves the robustness of DNNs by retraining them on a mixed set comprising both adversarial and clean samples. The experimental results indicate that AT can significantly reduce the forecast errors caused by white-box attacks, thus improving the robustness of the forecasting model. At present, adversarial defense strategies for wind power forecasting remain scarce, with only a limited number of methods reported in existing studies. Moreover, current research is almost exclusively confined to white-box defense scenarios, while black-box defense, which is more relevant to practical applications, remains largely unexplored.

**Fig 1:**
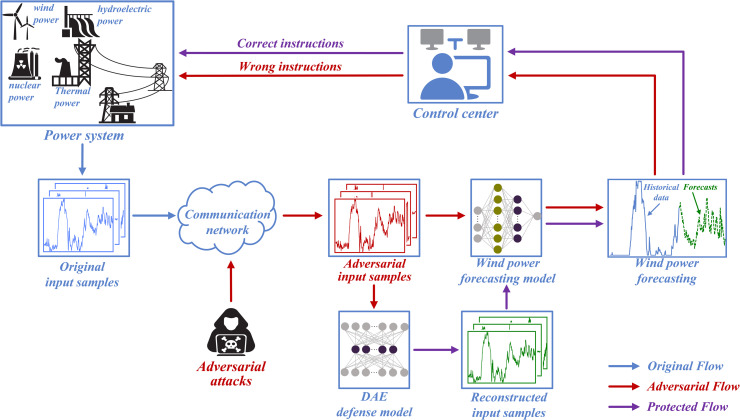
Adversarial attacks and defenses in wind power forecasting system.

Overall, research on adversarial attacks in wind power forecasting remains limited, and existing studies often overlook attack stealthiness. Most existing attack methods [25,27,30] typically perturb all input dimensions, which can degrade forecast accuracy but often at the cost of reduced attack stealthiness. To address this limitation, we design a dimension-constrained momentum iterative fast gradient sign method (DC-MI-FGSM) that selectively perturbs the input dimensions with the greatest gradient impact, thereby improving attack stealthiness while maintaining strong attack effectiveness. The proposed method further incorporates a momentum-based optimization mechanism to stabilize the update direction during iterations, enabling the generation of adversarial samples that are both destructive and difficult to detect. To defend against such attacks, we develop a denoising autoencoder (DAE)-based preprocessing defense strategy. Through denoising training, the DAE learns to map adversarial samples back to their corresponding clean representations, thereby effectively mitigating adversarial perturbations. This preprocessing-based defense not only reduces forecasting errors under adversarial attacks, but also preserves the original forecasting accuracy on clean data. The proposed attack and defense methods are systematically evaluated under both white-box and black-box scenarios. The white-box setting represents the worst-case attack condition, while the black-box setting reflects more realistic application environments. The framework of the entire process is illustrated in Fig 1. In scenarios where the wind power forecasting system is subjected to adversarial attacks, the generated adversarial input samples may mislead the forecasting model into producing inaccurate forecasts, potentially causing the control center to issue wrong instructions. Through the preprocessing operation of the DAE, these adversarial input samples can be reconstructed into their clean forms, enabling the control center to maintain correct decisions.

The primary contributions of this paper are outlined as follows:

1. A dimension-constrained attack, DC-MI-FGSM, is proposed for wind power forecasting. This method incorporates the momentum mechanism to stabilize the optimization direction of the attack while constraining the perturbation dimensions of the input samples, thereby generating effective and stealthy adversarial perturbations.
2. A DAE-based defense model is developed as a preprocessing strategy for wind power forecasting. Through an encoder–decoder architecture, the model denoises adversarial samples and reconstructs them into clean representations, thereby providing robust defensive performance.
3. The adversarial defense performance is systematically evaluated under both white-box and black-box attack scenarios. The defense model trained in the white-box setting maintains strong effectiveness against black-box attacks with varying perturbation strengths, demonstrating good generalization capability across diverse attack environments.
4. Comprehensive experimental comparisons are conducted under different attack settings. Compared with existing attack methods [25,30], DC-MI-FGSM induces more severe degradation in forecast accuracy while exhibiting higher stealthiness due to its dimension-constrained design. Moreover, compared with adversarial training [25], the proposed DAE-based defense more effectively reduces forecast errors while better preserving the original forecast accuracy.

The rest of this paper is organized as follows. Section 2 introduces the DNN-based wind power forecasting task and model, analyzes the attack environments and objectives, and provides a detailed description of the proposed attack algorithm. In section 3, the defense algorithm is formulated in detail. Section 4 validates the effectiveness of the proposed attack and defense algorithms through a series of experiments, with a detailed comparison to existing methods. Finally, Section 5 provides the conclusion of this paper.

## Formulation of forecast and attack

### Wind power forecasting

DNN-based wind power forecasting models typically utilize the historical wind power data and its influencing factors to predict the future wind power output. The historical dataset is generally divided into a training set and a testing set. The training set is denoted as ${𝒟}_{\text{tr}}=\{(x_{t-h},\ldots ,x_{t-1});P_{t+k}\overset{T_{\text{tr}}}{\underset{t=h}{\}}}$ where $x_{t-i}$ (1 < *i* < *h*, with *h* representing the length of historical data) denotes the input samples that include wind speed $\left(\overset{\text{ws}}{\underset{t-i}{x}}\right)$, wind direction $\left(\overset{\text{wd}}{\underset{t-i}{x}}\right)$, ambient temperature $\left(\overset{\text{Et}}{\underset{t-i}{x}}\right)$, and historical wind power data $\left(P_{t-i}\right)$, etc. *P*_*t*+*k*_ represents the future wind power output, and *k* is the forecasting horizon. The test set ${𝒟}_{\text{t}}=\{(x_{t-h},\ldots ,x_{t-1});P_{t+k}{\}}_{t=T_{\text{tr}}+h+1}^{T_{t}}$ consists of similar samples, which is used to assess the forecasting performance of the models.

During the training process, the mean absolute error (MAE) serves as the loss function *L*(*X*_*t*_) to quantify the wind power forecast errors, as follows:


$$L(X_{t})=\min_{\theta }\frac{1}{T_{tr}-h+1}\sum_{t=h}^{T_{tr}}|f_{\theta }(X_{t})-P_{t+k}|$$ (1)

where *T*_tr_ represents the number of training samples. The forecasting model $f_{\theta }$ describes the relationship between the input samples $X_{t}=(x_{t-h},\cdots ,x_{t-1})$ and the forecasted values *P*_*t*+*k*_ in the form of the model parameter set θ.

Through repeated training, the θ can be optimized to minimize the loss function, thus improving forecast accuracy. The optimization process is as follows:


$$\theta_{n}=\theta_{n-1}-\eta \cdot \nabla_{\theta }L(X_{t})$$ (2)

where η denotes the learning rate and $\nabla_{\theta }L(X_{t})$ represents the gradient of the loss function.

The LSTM can capture long-term dependencies through its gating mechanisms, making it highly effective for processing time series data [17]. This type of model has been widely employed in wind power forecasting and has demonstrated excellent performance (e.g., [13,17,32]). Existing studies [25] and [26] have also adopted LSTM to investigate adversarial security in wind power forecasting. Therefore, this work employs this mature and well-validated model to evaluate the performance of the proposed attack and defense strategies.

**Fig 2:**
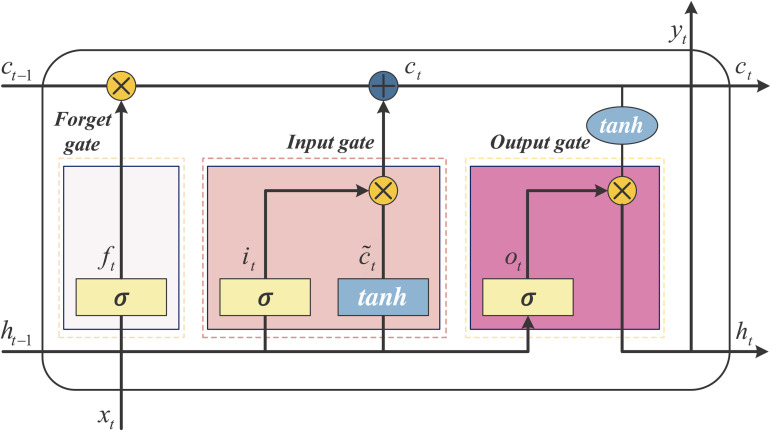
Structure of the LSTM layer.

As showed in Table 1, our forecasting model consists of an LSTM layer and multiple fully connected layers. The LSTM layer consists of a forget gate, an input gate, and an output gate, as shown in Fig 2. With the tuning of the forget gate, the LSTM can efficiently retain and propagate information on long sequences, thus capturing long-term dependencies. Given the input data *x*_*t*_, the cell state *c*_*t*_ processes the time series through the following process:


$$\left\{ \begin{array}{c} i_{t}=\sigma (W_{i}\cdot [h_{t-1},x_{t}]+b_{i})\\ o_{t}=\sigma (W_{o}\cdot [h_{t-1},x_{t}]+b_{o})\\ f_{t}=\sigma (W_{f}\cdot [h_{t-1},x_{t}]+b_{f})\\ c_{t}=f_{t}+i_{t}\cdot \tanh(W_{c}\cdot [h_{t-1},x_{t}]+b_{c})\\ h_{t}=o_{t}\cdot \tanh(c_{t}) \end{array}\right.$$ (3)

**Table 1:** Structure of the forecasting model.

Model type	Layer	Units	Activation	Optimizer
Forecasting model	LSTM	64	Tanh	Adam
	Dense	64	ReLU	
	Dense	32	ReLU	
	Dense	16	Tanh	
	Dense	1	Linear	

where *i*_*t*_ denotes the state of the input gate, *o*_*t*_ denotes the state of the output gate, and *f*_*t*_ denotes the state of the forget gate. *W*_*i*_, *W*_*o*_ and *W*_*f*_ are the weight matrices of the three gates, and *b*_*i*_, *b*_*o*_ and *b*_*f*_ are the corresponding bias vectors. σ represents the sigmoid activation function, and *h*_*t*_ denotes the hidden state. The number of LSTM units and the configuration of the fully connected layers are referenced to the general setup and adjusted through experiments, aiming to balance model complexity with forecasting accuracy.

### Attack environment and objective

Adversarial attacks are generally categorized into white-box and black-box attacks [33–35], depending on the attacker’s level of knowledge and access to the target system. In a white-box attack setting, the attacker has full knowledge of the forecasting model, including its architecture and parameters, and generates adversarial perturbations accordingly. This setting typically represents the worst-case attack scenario. In contrast, in a black-box attack setting, the attacker has only limited knowledge of the target model, which more closely reflects realistic attack conditions in practical applications. Evaluating both white-box and black-box scenarios allows us to assess model security under both worst-case and realistic threat models, thereby providing a more comprehensive security analysis framework. To simulate the black-box environment, adversarial perturbations are generated using a substitute model. Since employing the same model type can increase the attack success rate due to improved transferability, an LSTM-based model is adopted as the substitute model. This substitute model consists of an LSTM layer followed by multiple fully connected layers, and its detailed structure is summarized in Table 2.

**Table 2:** Structure of the substitute model.

Model type	Layer	Units	Activation	Optimizer
Substitute model	LSTM	64	Tanh	Adam
	Dense	64	Relu	
	Dense	32	Relu	
	Dense	32	Tanh	
	Dense	16	Tanh	
	Dense	1	Linear	

The objective of the attackers is to create adversarial samples ${\hat{X}}_{t}$ by introducing adversarial perturbations in the neighborhoods of the original input samples *X*_*t*_ under given constraints, thereby maliciously increasing or decreasing the wind power forecasts. Formally, the adversarial samples are generated by solving the following optimization problem:


$$\begin{array}{cc} & L(X_{t})=\max_{X_{t}}\gamma \cdot f(\hat{X_{t}})\\ & \text{subject to }\hat{X_{t}}=X_{t}+\delta_{X_{t}}\\ & \;\|\delta_{X_{t}}{\|}_{p}\leq \epsilon \end{array}$$ (4)

where $\delta (X_{t})$ represents the adversarial perturbation, and ϵ denotes the perturbation strength. The γ denotes the adjustment factor: when γ=−1, attackers attempt to optimize *X*_*t*_ to maliciously decrease the wind power forecasts, when γ=1, the goal is to maliciously increase the forecasts. To evade anomaly detection system, it is necessary to constrain the perturbation range $\|\delta {\|}_{p}$, where *p* can be set to 0, 1, or ∞ to correspond to different perturbation constraints [22,31,36].

The objective and optimization direction of problem (4) are opposite to those of problem (2). For problem (2), the forecasting task optimizes the model parameter set θ through gradient descent to minimize forecast errors. In contrast, for problem (4), the attack task optimizes the input samples *X*_*t*_ through gradient ascent to maximize forecast errors.

### Proposed attack algorithm

FGSM [31] is a single-step attack method that uses the gradient of the optimization objective in Eq (4) to perform a one-step update for generating adversarial perturbations. However, single-step attack only performs one gradient update and cannot iteratively optimize perturbations, often resulting in poor adversarial samples. MI-FGSM [37] is a variant of FGSM that employs a multi-step iterative optimization strategy. By incorporating momentum into each iteration, MI-FGSM ensures stable gradient updates and escapes from poor local optima, thereby generating more effective adversarial samples.

The MI-FGSM attack first uses the momentum term *g* to accumulate gradient information from the previous n−1 iterations and the current iteration. Then, a momentum decay factor μ stabilizes the optimization direction. During each iteration, the perturbation is updated using a step size ϵ/N, and the gradient is normalized using its *L*_1_ norm. The detailed computational procedure is as follows:


$$\begin{array}{cc} & g_{n}=\mu \cdot g_{n-1}+\frac{\nabla_{X_{t}}L({\hat{X}}_{t,n})}{\|\nabla_{X_{t}}L({\hat{X}}_{t,n}){\|}_{1}}\\ & \delta_{n}=\frac{\epsilon }{N}\cdot \text{sign}(g_{n})\\ & \text{subject to }\|\delta_{n}{\|}_{\infty }\leq \epsilon \\ & X_{t,n}=X_{t,n-1}+\delta_{n} \end{array}$$ (5)

where *N* denotes the number of iterations and sign(·) represents the sign function. The constraint $\|\cdot {\|}_{\infty }$ indicates that each element of the input samples is perturbed independently with a limited magnitude. With the momentum optimization mechanism, MI-FGSM generates adversarial samples that cause greater forecast errors while keeping the overall perturbation magnitude relatively small due to varying update directions.

While the momentum mechanism in MI-FGSM is effective for optimizing perturbations, its $L_{\infty }$-norm constraint results in perturbations being indiscriminately applied to all input data, which may make the attack easier to detect. Inspired by previous research [38,39], the *L*_0_ constraint offers an effective strategy by only attacking the most influential input dimensions—those with the greatest impact on the gradient. To this end, we propose DC-MI-FGSM, an attack that employs an *L*_0_-norm constraint to enhance stealth by limiting the number of perturbed dimensions.

To achieve this, a new attack constraint $\left\|\delta {\|}_{0}\leq \tau |X_{t}\right|$ is constructed, where |*X*_*t*_| represents the number of dimensions of the input samples, and τ denotes the tampering proportion. The new attack constraint satisfies the *L*_0_-norm restriction, indicating that attackers only need to tamper with the $\tau |X_{t}|$ input dimensions that have the largest impact on the gradient $\nabla_{X_{t}}L({\hat{X}}_{t})$ to achieve an effective attack. The generation of the adversarial samples and the corresponding perturbation constraint are expressed as follows:


$$\begin{array}{cc} & {\hat{X}}_{t,n}={\hat{X}}_{t,n-1}+\delta_{n}^{A}\\ & \text{subject to }\delta_{n}^{S⧵A}=0 \end{array}$$ (6)

where *A* represents the set of tampered input samples, *S* denotes the full set of input samples, and S⧵A represents the complement of *A* in *S*.

**Algorithm 1. The DC-MI-FGSM attack against wind power forecasting.**



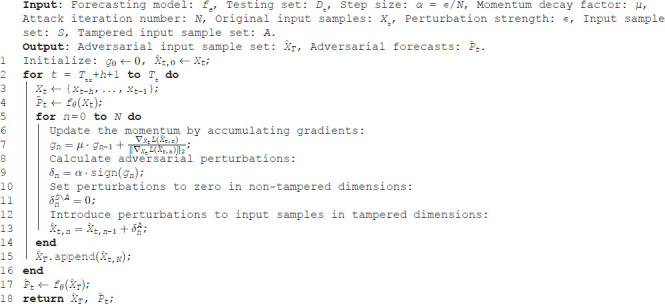



The implementation of DC-MI-FGSM is shown in Algorithm 1. The initialization of this algorithm includes *g*_0_ = 0 and ${\hat{X}}_{t,0}=X_{t}$. After *N* iterations, the algorithm generates the final adversarial sample ${\hat{X}}_{t,N}$.

### Proposed defense method

#### Motivation.

Adversarial attacks aim to deceive DNN models by applying carefully crafted, subtle perturbations to input samples. To ensure the secure application of DNNs, an effective strategy is to remove these perturbations through preprocessing, restoring the original, clean samples [28]. At present, defense research in wind power forecasting has only explored adversarial training [25] and data replacement defense [30]. The defense mechanisms based on preprocessing strategies have not yet been investigated. Therefore, this paper explores a DAE defense model based on preprocessing strategy to defend against adversarial attacks in wind power forecasting.

Adversarial perturbations can be viewed as a form of noise contamination, and effective denoising preprocessing strategies can be used to defend against adversarial attacks. In the field of semantic segmentation, DAE is used to remove adversarial perturbations and restore the original image, thereby improving the performance of segmentation models under attacks [40]. DAE is also applied in power quality disturbance classification (PQD) [41]. Through denoising, adversarial PQD signals are restored to clean signals to ensure accurate classification. Inspired by these works, this paper proposes a deep learning defense model based on DAE tailored to process the attacked data for wind power forecasting. This method not only offers robust protection but also ensures the forecast accuracy.

### DAE defense model

DAE is a variant of autoencoder (AE) designed to reconstruct data corrupted by noise [42]. Through denoising training, DAE can map adversarial samples (viewed as “noisy” inputs) back to their clean forms [43]. Unlike classification tasks, forecasting tasks such as wind power forecasting involve time-dependent data, which requires the DAE defense model to be specifically designed to accommodate temporal sequences.

**Fig 3:**
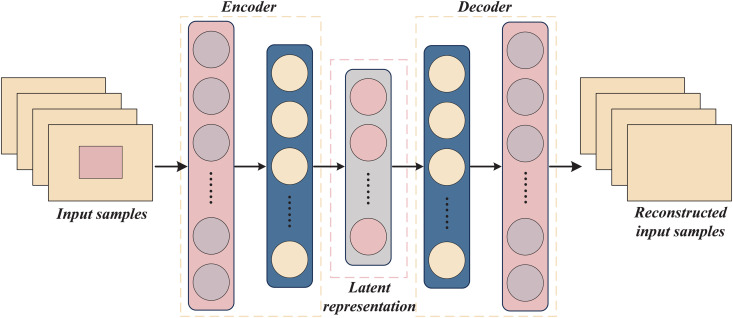
Structure of the DAE defense model.

As Fig 3 shows, the DAE consists of an encoder and a decoder, and its specific structure for this study is provided in Table 3. The encoder includes an LSTM layer followed by Dense layers. The LSTM is employed to capture temporal dependencies within the wind power time-series data, while the dense layer transforms the LSTM’s output into a lower-dimensional representation. The encoder compresses the input samples into the latent representation: ${\mathbb{R}}^{2KL}\to {\mathbb{R}}^{z}$, achieved through the following nonlinear transformation:


$$q=\sigma (W_{1}\cdot X_{DAE}+b_{1})$$ (7)

**Table 3:** Structure of DAE.

Model type	Layer	Units	Activation	Optimizer
Defense model	LSTM	128	Tanh	Adam
	Dense	64	Linear	
	Dense	32	Linear	
	LSTM	64	Tanh	
	Dense	128	Linear	
	Dense	9	Linear	

where $q\in {\mathbb{R}}^{z}$ denotes the latent representation of the input sample, σ denotes the activation function, *W*_1_ represents the weight matrix, *b*_1_ represents the bias vector, and *X*_DAE_ denotes the input of the DAE.

The decoder consists of an LSTM layer followed by dense layers. The LSTM layer is responsible for reconstructing the sequence and further reducing noise. The decoder reconstructs the data from the latent representation back to the original input space: ${\mathbb{R}}^{z}\to {\mathbb{R}}^{2KL}$, involving the following process:


$$\tilde{X}=\sigma (W_{2}\cdot q+b_{2})$$ (8)

where $\tilde{X}\in {\mathbb{R}}^{2KL}$ represents the reconstructed data, *W*_2_ represents the weight matrix, and *b*_2_ represents the bias vector.

During the training phase, the reconstruction error between the reconstructed and original samples is quantified using the mean squared error (MSE), calculated as follows:


$$L(\tilde{X})=\min_{D}\frac{1}{n}\sum_{i=0}^{n}{\left(X_{target,i}-{\tilde{X}}_{i}\right)}^{2}$$ (9)

where *n* denotes the size of the training dataset and *X*_target, *i*_ represents the expected clean output of the DAE.

The implementation of the DAE for wind power forecasting is detailed in Algorithm 2. The training set consists of *X*_DAE_ and *X*_target_. To maintain the original forecasting accuracy, the DAE should accurately reconstruct the original input samples as much as possible. Therefore, all original input samples *X*_*t*_ need to be included in the training set, denoted as $X_{\text{DAE}}\leftarrow \text{concatenate}({\hat{X}}_{t},X_{t})$ and $X_{\text{target}}\leftarrow \text{concatenate}(X_{t},X_{t})$. This concatenation allows the DAE to learn the distinction between noisy and clean inputs, thereby enhancing their robustness against adversarial while retaining forecast accuracy.

**Algorithm 2. Construction of the DAE defense for wind power forecasting**



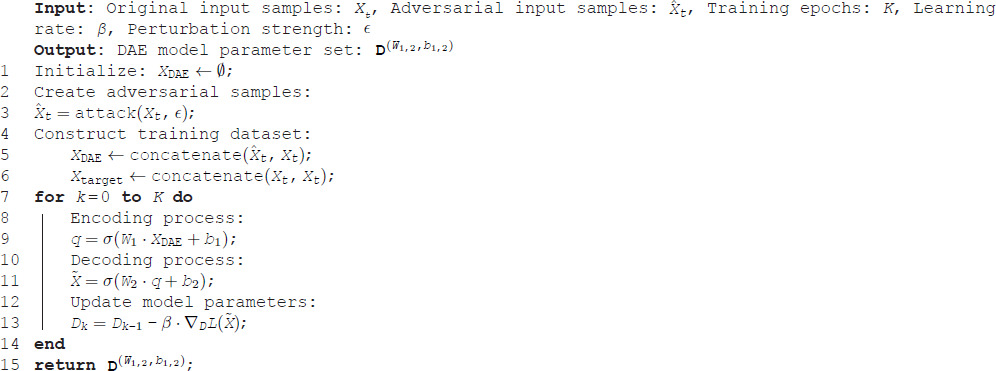



## Case studies

### Dataset description and experimental setup

This study employs the Spatial Dynamic Wind Power Forecasting (SDWPF) dataset [44], provided by China Longyuan Power Group Co., Ltd. The SDWPF dataset contains data from 134 wind turbines, with each turbine’ s data independently collected at a recording resolution of 10 minutes. This dataset encompasses over 245 days of data, including factors such as temperature, wind speed, and wind direction. Based on previous research, to evaluate the performance of the proposed methods, a wind turbine is randomly selected to evaluate the performance of the proposed attack and defense methods.

The original data is resampled to a 1-hour resolution by averaging every six records [45]. All input samples are normalized to the [0,1] range to enhance model convergence and stability. Of the entire dataset, 80% is used as the training set, while the remaining 20% serves as the testing set. The learning rate is initially set to 0.01, and the number of training epochs is set to 80. To prevent overfitting, techniques such as Dropout and *L*_2_ regularization are applied. The Adam optimizer is employed to train the model. A callback function is used to optimize both the learning rate and the number of training epochs. Specifically, if the loss value does not decrease over five consecutive iterations, the learning rate is reduced to one-tenth of its current value, and if the loss value does not decrease for 10 consecutive iterations, training is terminated early. All reported performance metrics are averaged over five independent experimental runs to mitigate the effects of stochasticity in model training and initialization. The experiments are conducted using TensorFlow and Keras in the environment equipped with NVIDIA GeForce RTX 3050 GPUs.

### Momentum decay factor setting

The DC-MI-FGSM attack algorithm generates adversarial samples by applying the momentum optimization mechanism during the attack iteration. The momentum decay factor μ is a key parameter that stabilizes the direction of the gradient update, thereby generating high-quality adversarial samples. However, when μ=0, DC-MI-FGSM loses its momentum effect and degenerates into the regular iterative attack, which may lead to overfitting of the adversarial samples and consequently degrade the attack performance.

**Fig 4:**
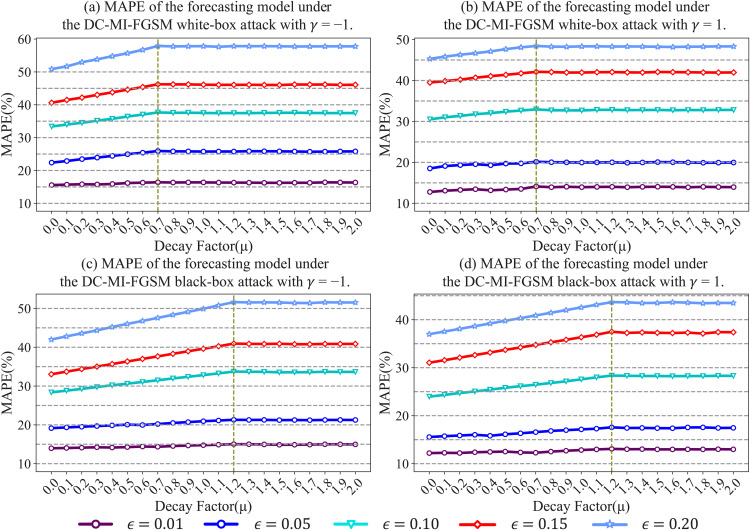
Performance of the DC-MI-FGSM attack algorithm under different momentum decay factors across different attack scenarios.

To determine the optimal decay factors in both white-box and black-box environments, the attack experiments are conducted on the testing set. The decay factor μ is incrementally varied from 0 to 2 with a step size of 0.1, the perturbation strength ϵ is set to {−0.2,−0.15,−0.1,−0.05,−0.01,0.01,0.05,0.1,0.15,0.2}, and the number of attack iterations *N* is set to 50. Fig 4 illustrates the attack performance under different momentum decay factors, with the mean absolute percentage error (MAPE) as the evaluation metric. As shown in Fig 4(a) and 4(b), under different attack directions (γ=±1) and perturbation strengths, the MAPE under white-box attacks reaches its maximum value when μ=0.7. Fig 4(c) and 4(d) show the MAPE under black-box attacks, where the MAPE attains its maximum value at μ=1.2 across different attack directions and perturbation strengths. Therefore, μ is set to 0.7 in the white-box environment and 1.2 in the black-box environment.

### Analysis and comparison under white-box attacks

**Fig 5:**
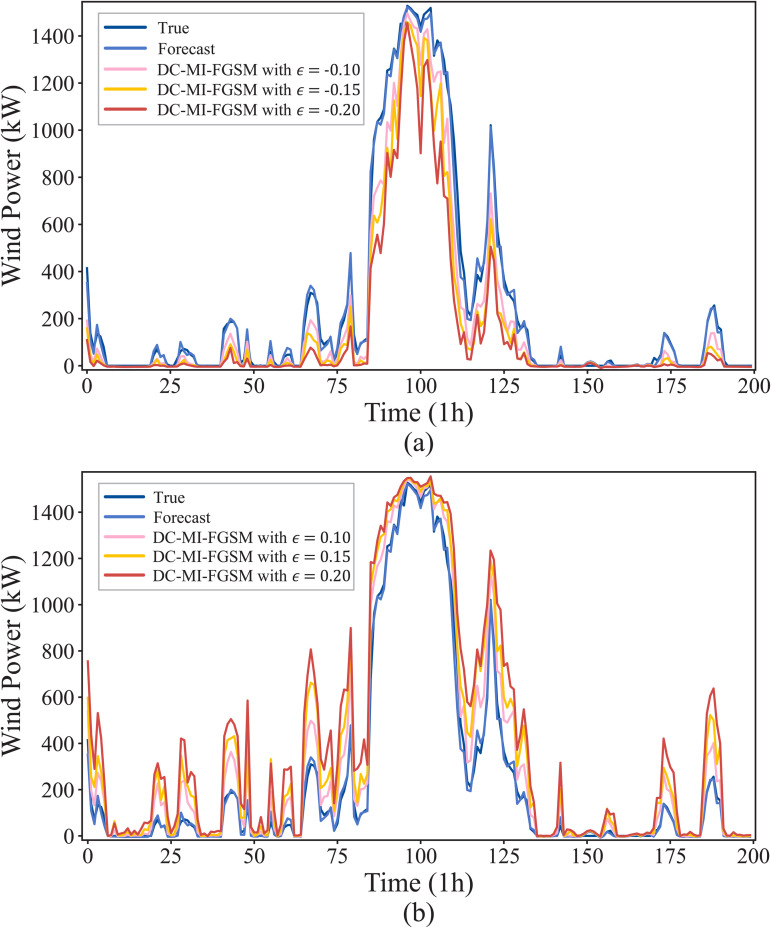
Adversarial attacks on wind power forecasting under the white-box scenario. (a) Impact of DC-MI-FGSM (ϵ={−0.2,−0.15,−0.1}) on the forecast curves when γ=−1. (b) Impact of DC-MI-FGSM (ϵ={0.1,0.15,0.2}) on the forecast curves when γ=1.

In the white-box environment, attackers perform the DC-MI-FGSM attack by fully utilizing the training parameters and structure of the forecasting model. To visually show the effects of the DC-MI-FGSM white-box attacks, Fig 5 illustrates the wind power forecast curves under different attack directions and perturbation strengths. As shown in Fig 5(a), when *γ* = −1, by injecting adversarial samples with perturbation strengths of {−0.2,−0.15,−0.1} into the wind power forecasting model, the forecasted values can be maliciously decreased, resulting in the forecast curves to deviate downward from the original curve. As the perturbation strength increases, the attacked forecast curves deviate more obviously. In Fig 5(b), when γ=1, attackers maliciously increase the wind power forecasts by introducing perturbations with strengths of {0.1, 0.15, 0.2}. This causes the forecast curves to deviate upward from the original curve, with the deviation becoming more pronounced as the perturbation strength increases. In addition, these attacked forecast curves can well track the dynamics of the original curve, meaning the attacks may evade detection by both human eyes and monitoring systems.

In order to demonstrate the advantages of the DC-MI-FGSM attack, we compare it with the FGSM and PGD in terms of attack effectiveness and stealthiness. The corresponding parameters are set as follows: (1) For FGSM, the perturbation strengths ϵ are set to {−0.2,−0.15,−0.1,−0.05,−0.01,0.01,0.05,0.1,0.15,0.2}. The upper bound of 0.2 ensures perturbations remain realistic, thereby preventing unrealistic forecast errors. (2) For PGD, ϵ is set to {−0.2,−0.15,−0.1,−0.05,−0.01,0.01,0.05,0.1,0.15,0.2}, and the number of attack iteration *N* is set to 50, which is sufficient for algorithm convergence. (3) For DC-MI-FGSM, μ is set to 0.7, ϵ is set to {−0.2,−0.15,−0.1,−0.05,−0.01,0.01,0.05,0.1,0.15,0.2}, and *N* is set to 50. To achieve a substantial impact on forecast accuracy while ensuring a high level of stealthiness, the perturbation dimension $\tau |X_{t}|$ is set to 60.

For the assessment of attack effectiveness, MAPE, RMSE and MAE are used as the evaluation metrics to quantify forecast errors under attacks. Table 4 reports the forecasting errors in the white-box setting, where the most severe errors in each case are highlighted in bold. As shown in Table 4, across all attack directions and perturbation strengths, DC-MI-FGSM consistently causes larger forecasting errors than FGSM. In most cases, DC-MI-FGSM also outperforms PGD, with only a few exceptions where PGD exhibits slightly stronger effects. This is because DC-MI-FGSM adopts an *L*_0_-norm constraint that concentrates perturbations on the input dimensions with the greatest impact on the gradient, and a momentum-based optimization strategy that stabilizes the attack direction during iterations. PGD and FGSM employ the $L_{\infty }$-norm constraint to limit the magnitude of perturbations across all input dimensions. Therefore, the proposed attack is more effective than existing methods in degrading forecasting performance.

**Table 4:** Forecast errors of different attack methods under varying perturbation strengths in the white-box environment.

White-box attacks	Perturbation strength	MAPE (%)		RMSE (kW)		MAE (kW)	
		γ=−1	γ=1	γ=−1	γ=1	γ=−1	γ=1
No attack	–	12.02	12.02	76.64	76.64	44.59	44.59
DC-MI-FGSM	ϵ = 0.01	14.12	16.38	83.73	87.16	47.51	48.45
	ϵ = 0.05	**20.15**	**25.93**	**91.32**	**100.63**	**54.31**	**59.74**
	ϵ = 0.10	**32.95**	37.67	**120.83**	134.81	**67.34**	84.18
	ϵ = 0.15	**42.11**	**46.24**	**158.71**	**182.27**	**90.56**	**114.57**
	ϵ = 0.20	**48.44**	**57.91**	**198.17**	**240.43**	**120.63**	**165.64**
FGSM	ϵ = 0.01	12.61	15.26	82.67	85.91	46.36	47.38
	ϵ = 0.05	17.94	21.82	87.61	95.89	51.11	55.92
	ϵ = 0.10	28.93	32.45	107.42	122.71	58.99	76.54
	ϵ = 0.15	37.82	40.38	135.06	156.74	77.36	102.19
	ϵ = 0.10	44.18	50.34	166.07	205.93	96.12	132.62
PGD	ϵ = 0.01	**15.14**	**16.52**	**85.49**	**88.36**	**47.14**	**48.96**
	ϵ = 0.05	19.86	25.77	90.05	98.83	49.74	57.79
	ϵ = 0.10	31.61	**37.82**	111.31	**136.04**	62.64	**87.74**
	ϵ = 0.15	40.75	45.34	144.53	171.96	83.05	109.42
	ϵ = 0.20	46.27	56.07	175.78	223.82	105.65	146.67

The attack stealthiness is a crucial factor in evaluating the attack performance, as larger perturbations to input samples may increase the risk of detection [46,47]. To assess the stealthiness, a new metric called average perturbation percentage (APP) is proposed to quantify the perturbation amplitude of attacks on input samples. The APP is computed as follows:


$$APP=\frac{1}{N\cdot d}\sum_{i=1}^{d}\sum_{j=1}^{N}\left|\frac{\overset{j}{\underset{i}{\hat{X}}}-\overset{j}{\underset{i}{X}}}{\overset{j}{\underset{i}{X}}}\right|$$ (10)

where $X_{i}^{j}$ represents the original sample, ${\hat{X}}_{i}^{j}$ represents the adversarial sample, with *i* representing the sample dimension and *j* the sample index. *N* is the number of samples, and *d* represents the number of sample dimensions. This metric considers that the perturbations from different dimensions may affect the forecast results to varying degrees. Therefore, it calculates the weighted sum of perturbation percentages for each dimension, rather than the simple average.

**Fig 6:**
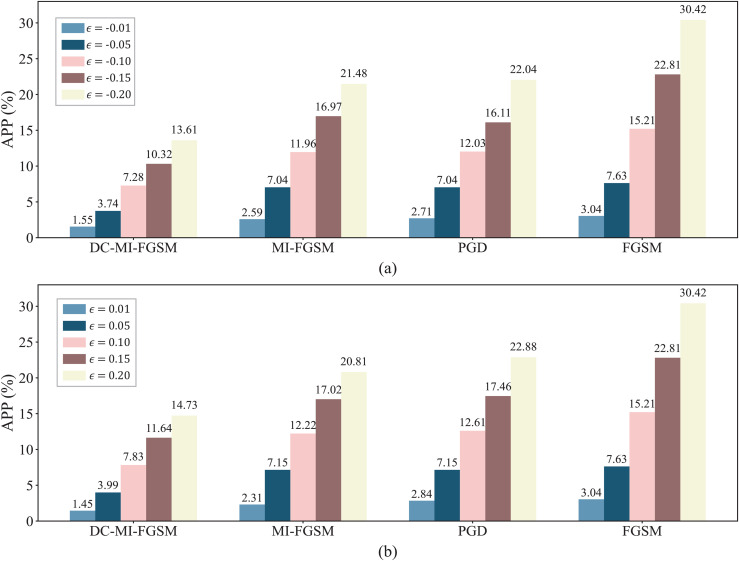
Stealthiness comparison of DC-MI-FGSM, FGSM, PGD, and MI-FGSM in the white-box environment. (a) APP of adversarial samples under different attacks when γ=−1. (b) APP of adversarial samples under different attacks when γ=1.

Fig 6 illustrates the APP of input samples under white-box attacks. Under different attack direction and perturbation strength, FGSM consistently exhibits the largest APP values, while MI-FGSM and PGD show comparable APP levels. In contrast, the proposed DC-MI-FGSM introduces significantly smaller APP values. This indicates that the proposed attack method achieves substantially improved stealthiness compared with existing attack methods. This is because the momentum optimization mechanism of our method adjusts the update direction in each iteration, which keeps the overall perturbation magnitude relatively small. In addition, its *L*_0_ norm constraint restricts the perturbed input dimensions, further enhancing the attack’s stealthiness.

By analyzing and comparing the experimental results in the white-box environment, it can be concluded that DC-MI-FGSM causes the forecast curves to deviate from the original curve, while effectively tracking its dynamics. Compared to FGSM and PGD, DC-MI-FGSM leads to more substantial increase in each forecast error metric, thereby more significantly degrading forecast accuracy. Moreover, DC-MI-FGSM generates obviously smaller perturbations to input samples than FGSM and PGD, and it also outperforms MI-FGSM in terms of stealthiness by constraining the perturbation dimensions.

### Analysis and comparison under black-box attacks

**Fig 7:**
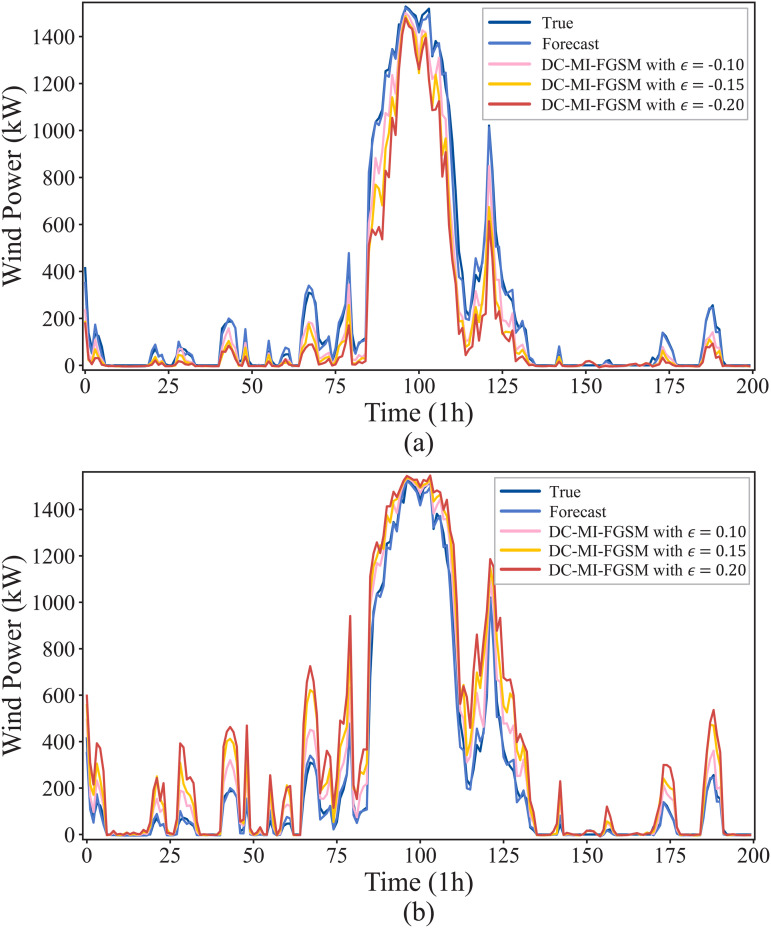
Adversarial attacks on wind power forecasting under the black-box scenario. (a) Impact of DC-MI-FGSM (ϵ={−0.2,−0.15,−0.1}) on the forecast curves when γ=−1. (b) Impact of DC-MI-FGSM (ϵ={0.1,0.15,0.2}) on the forecast curves when γ=1.

In the black-box environment, attackers often lack access to the training parameters and structure of the model, making black-box attacks the most realistic type of attacks in practical applications [35]. To perform the DC-MI-FGSM black-box attacks, the adversarial samples generated by the substitute forecasting model are used to attack the original forecasting model. Fig 7 illustrates the wind power forecast curves with perturbation strengths of {−0.2,−0.15,−0.1,0.1,0.15,0.2}. As shown in Fig 7(a), when γ=−1, the forecast curves deviate downward from the original curve, with the deviations becoming more pronounced as the perturbation strength increases. Fig 7(b) shows that when γ=1, the forecast curves deviate upward from the original curve, with the deviations becoming more noticeable as the perturbation strength rises. These results demonstrate that the DC-MI-FGSM black-box attacks effectively degrade forecast accuracy, highlighting the strong black-box transferability of the DC-MI-FGSM adversarial samples. Additionally, the success of the attack confirms the potential vulnerabilities of DNNs to black-box attacks, which may pose a genuine threat in practical, real-world scenarios.

For method comparison in the black-box environment, the parameter settings are the same as those in Section 4.3, except that the momentum decay factor of DC-MI-FGSM is set to 1.2. The impacts of DC-MI-FGSM, FGSM and PGD on forecast errors are shown in Table 5. As shown in Table 5, a trend similar to that observed in the white-box setting is also evident in the black-box environment. Under the different attack direction and perturbation strength, the proposed method outperforms FGSM in all cases and surpasses PGD in most scenarios. The result indicates that the adversarial samples generated by DC-MI-FGSM exhibit stronger black-box transferability, which lead to more significant forecast errors. However, comparing with Table 4, it can be observed that black-box attacks are less effective than white-box attacks under the different attack direction and perturbation strength. This is due to the differences of the training parameters and structure between the substitute model and the original model.

**Table 5:** Forecast errors of different attack methods under varying perturbation strengths in the black-box environment.

Black-box attacks	Perturbation strength	MAPE (%)		RMSE (kW)		MAE (kW)	
		γ=−1	γ=1	γ=−1	γ=1	γ=−1	γ=1
DC-MI-FGSM	ϵ = 0.01	13.08	**15.03**	82.73	**86.36**	45.33	**47.64**
	ϵ = 0.05	**17.57**	21.30	**89.44**	96.33	**49.31**	56.26
	ϵ = 0.10	28.35	**33.73**	112.02	**124.28**	61.42	**76.72**
	ϵ = 0.15	**37.49**	**40.94**	**138.42**	**166.43**	**79.11**	**106.25**
	ϵ = 0.20	**43.66**	**51.63**	**169.34**	**208.04**	**95.17**	**135.20**
FGSM	ϵ = 0.01	12.21	14.06	81.38	84.23	45.57	46.73
	ϵ = 0.05	15.79	19.67	86.21	90.09	48.02	53.51
	ϵ = 0.10	24.47	29.07	101.86	114.76	54.52	69.84
	ϵ = 0.15	32.97	35.49	124.37	143.58	69.18	90.24
	ϵ = 0.20	38.67	44.88	149.71	176.94	85.15	113.64
PGD	ϵ = 0.01	**13.89**	14.96	**84.57**	85.75	**46.81**	47.27
	ϵ = 0.05	17.41	**21.55**	87.26	**97.31**	48.22	**56.58**
	ϵ = 0.10	**28.78**	33.38	**115.25**	116.62	**62.29**	74.17
	ϵ = 0.15	36.07	38.96	126.27	158.42	74.38	101.37
	ϵ = 0.20	41.42	49.26	152.62	184.62	88.26	124.63

**Fig 8:**
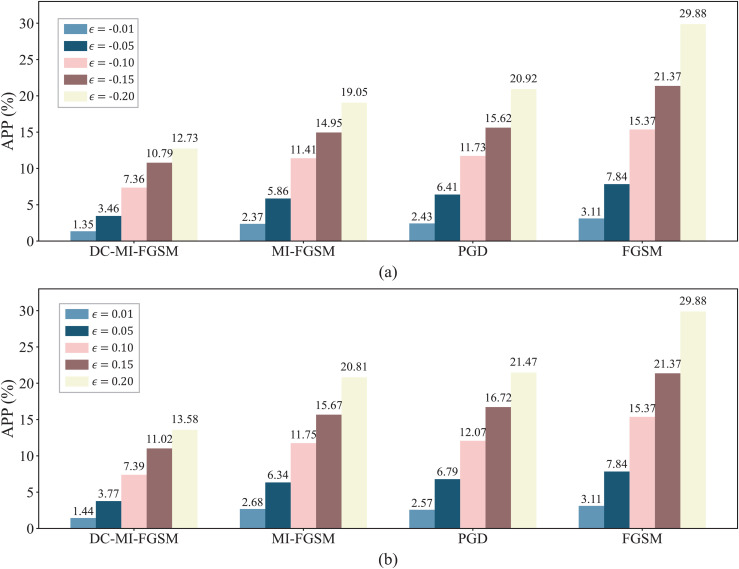
Stealthiness comparison of DC-MI-FGSM, FGSM, PGD, and MI-FGSM in the black-box environment. (a) APP of adversarial samples under different attacks when γ=−1. (b) APP of adversarial samples under different attacks when γ=1.

To assess the attack stealthiness, Fig 8 compares the APP of input samples under different black-box attacks. As shown in the figure, the APP produced by DC-MI-FGSM is obviously smaller than that by FGSM, PGD and MI-FGSM under the different attack direction and perturbation strength. This indicates that DC-MI-FGSM remains significantly more stealthy than FGSM, PGD and MI-FGSM in the black-box environment.

By analyzing and comparing the experimental results in the black-box environment, it can be concluded that DC-MI-FGSM also causes the forecast curves to deviate from the original curve, leading to inaccurate forecasts. Compared to FGSM and PGD, DC-MI-FGSM induces greater forecast errors, indicating its stronger black-box transferability. Additionally, DC-MI-FGSM generates smaller perturbations to input samples than FGSM and PGD, highlighting its excellent stealthiness.

### Defense performance under white-box attacks

In order to evaluate the performance of the DAE defense against the DC-MI-FGSM white-box attacks under different perturbation strengths and directions, four schemes are designed to train the DAE, allowing us to determine the most suitable training configuration. The common parameters for all schemes include the SGD optimizer, the learning rate of 0.001, and the training epoch of 50. The schemes are distinguished by the perturbation strength of the adversarial samples used for training. Specifically, DAE-1 is trained with a strong negative perturbation (ϵ=−0.2), and DAE-2 with a strong positive perturbation (ϵ=0.2). To assess the effectiveness of training with weaker perturbations, DAE-3 is trained with a moderate negative perturbation (ϵ=−0.1), and DAE-4 with a moderate positive perturbation (ϵ=0.1).

**Fig 9:**
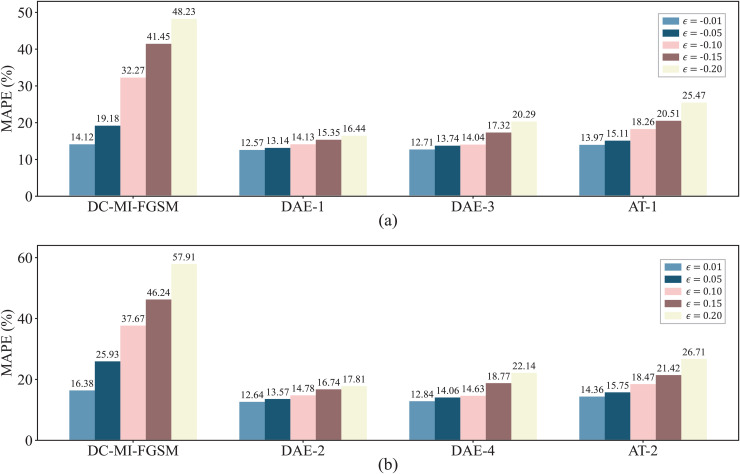
Comparison of defense performance between DAE and AT in the white-box environment. (a) MAPE reduction of the attacked forecasting model under different defense strategies when γ=−1; (b) MAPE reduction of the attacked forecasting model under different defense strategies when γ=1.

For comparing the defense algorithms in the white-box environment, two AT training schemes for coping with attacks from different directions are established: AT-1 (γ=−1) and AT-2 (γ=1). The parameters are set as follows: the perturbation strengths of the adversarial samples used for training are set to ±0.2, the number of training epochs is set to 80, and the optimizer is set to Adam. The comparison results are shown in Fig 9. It is clearly observed that DAE significantly reduces forecast errors under adversarial attacks, effectively mitigating the impact of such attacks. Compared with AT, DAE achieves greater reductions in post-attack forecast errors, indicating its superior defensive performance in wind power forecasting. Furthermore, DAE-1 and DAE-2 consistently outperform DAE-3 and DAE-4 in reducing forecast errors after attacks, reflecting stronger defensive capabilities. This improvement can be attributed to the fact that training with higher-strength perturbations can enhance the DAE’s defense capability against various perturbation strengths.

**Fig 10:**
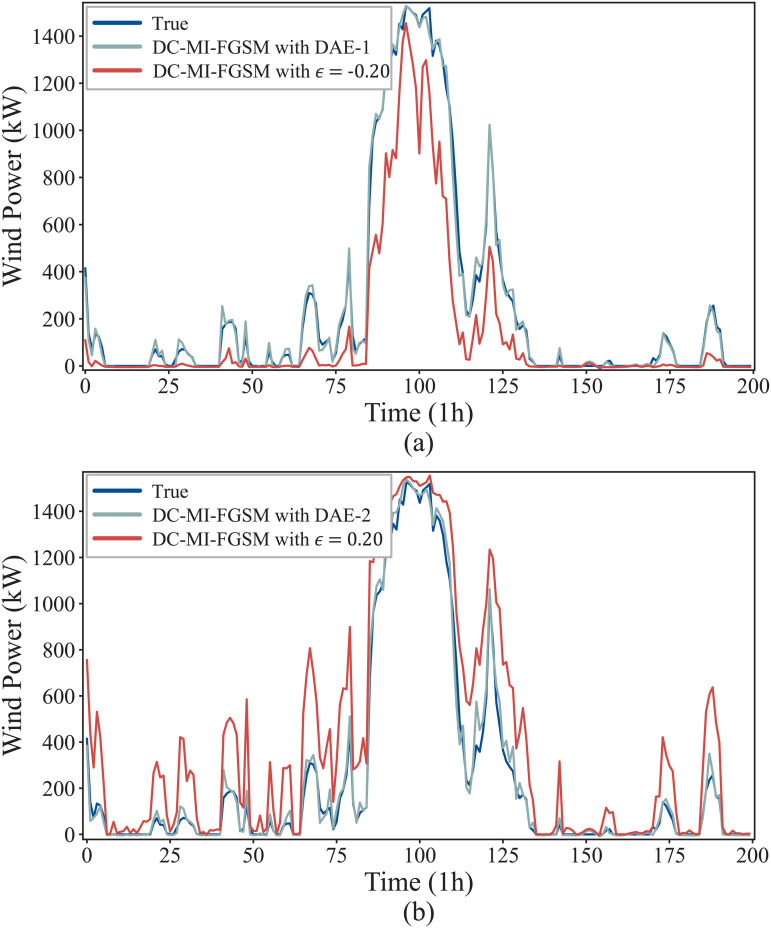
Defense performance of DAE against the DC-MI-FGSM white-box attacks. (a) Restoration of the attacked forecast curve under the DAE defense when γ=−1. (b) Restoration of the attacked forecast curve under the DAE defense when γ=1.

To visualize the defense effectiveness of DAE, Fig 10 shows the forecast curves under its defense against the DC-MI-FGSM white-box attacks. For both attack directions, the attack causes a noticeable deviation in the forecast curves from the original curve. Through the preprocessing operation of the DAE, the wind power forecasts maliciously decreased or increased by DC-MI-FGSM can be restored very well. These results demonstrate that the DAE can effectively defense against the DC-MI-FGSM white-box attacks.

For comparing the impacts of DAE and AT on the original forecast accuracy, the forecasting model equipped with defense algorithms is used to predict the original input samples, and the forecast errors are shown in Table 6. After preprocessing by DAE-1, DAE-2, DAE-3, DAE-4, the MAPE increased by 0.44%, 0.46%, 0.42% and 0.50% respectively, while after implementing AT-1 and AT-2, the MAPE increased by 1.61% and 1.68%, respectively. These results indicate that DAE more effectively retains the original forecast accuracy in the absence of perturbations than AT.

**Table 6:** Forecast errors of the forecasting model with defense algorithms.

Evaluation metric	None	DAE-1	DAE-2	AT-1	AT-2
MAPE (%)	12.02	12.46	12.48	**13.63**	**13.70**

By analyzing and comparing the experimental results in the white-box environment, it can be concluded that the DAE exhibits effective defense performance, obviously restoring the attacked forecast curves. Moreover, DAE is more effective than AT in reducing the forecast errors caused by the DC-MI-FGSM white-box attacks and performs better in preserving the original forecast accuracy.

### Defense performance under black-box attacks

**Fig 11:**
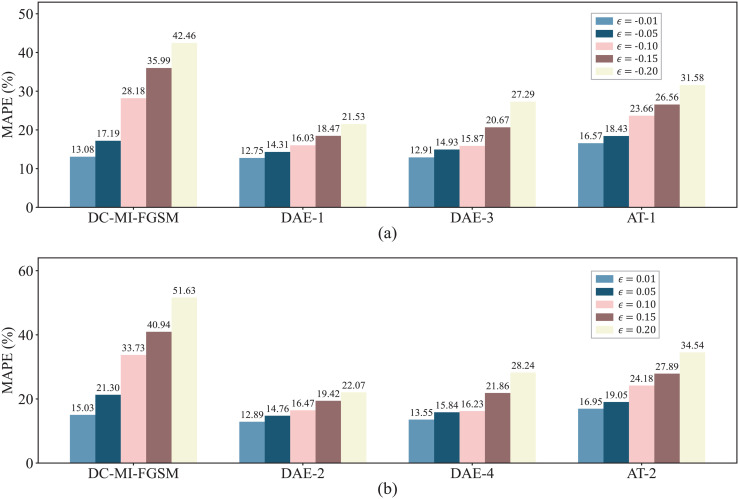
Comparison of defense performance between DAE and AT in the black-box environment. (a) MAPE reduction of the attacked forecasting model under different defense strategies when γ=−1; (b) MAPE reduction of the attacked forecasting model under different defense strategies when γ=1.

In order to evaluate the performance of the DAE defense algorithm in the black-box environment, we use the DAEs trained in the white-box setting to resist black-box attacks. Fig 11 compares the performance of different defense algorithms against the DC-MI-FGSM black-box attacks. It is evident that, under different attack directions and perturbation strengths, the DAE consistently outperforms AT, achieving more substantial reductions in forecast errors. This indicates that DAE trained in a white-box setting generalize more effectively to black-box attacks than the existing method AT. However, as observed in Figs 11 and 9, the performance of the DAE in the black-box environment is weaker than in the white-box environment. This is because the DAE is trained in the white-box environment, while the adversarial samples generated by the DC-MI-FGSM black-box attacks are not included in the training set, thereby reducing the performance of the DAE in the black-box environment. Additionally, it is worth noting that the AT fails to defend against the DC-MI-FGSM black-box attacks at lower perturbation strengths (ϵ=0.01, −0.01, and −0.05). In these cases, AT does not mitigate the attack impact but instead further aggravates the forecast errors. These results demonstrate that the DAE maintains its robustness even against attacks where AT fails to provide effective defense.

**Fig 12:**
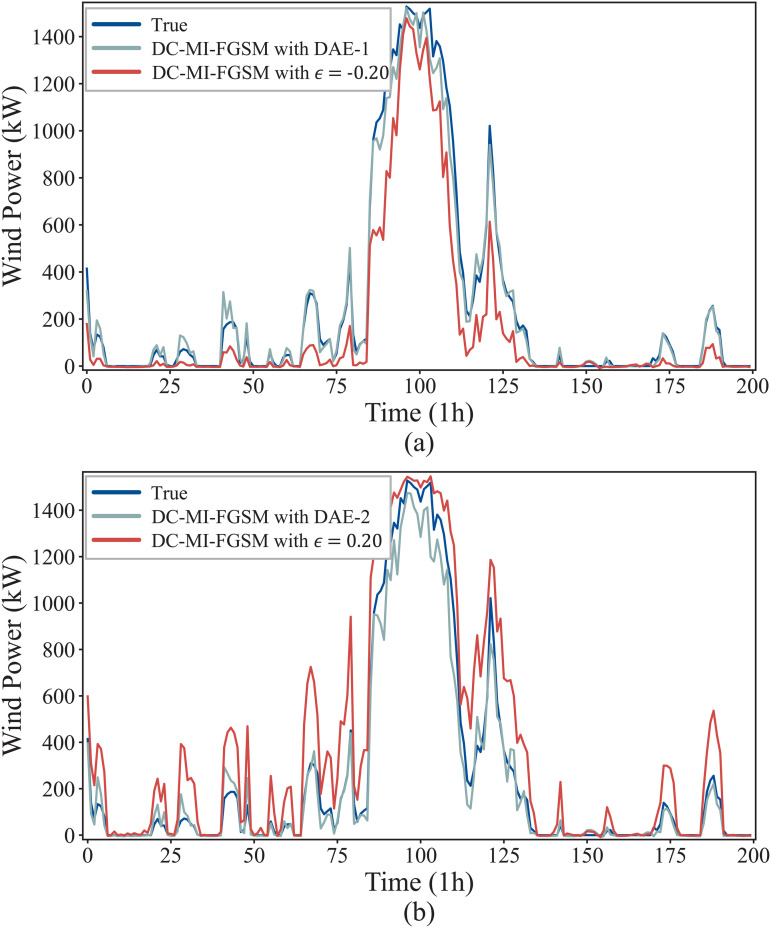
Defense performance of DAE against the DC-MI-FGSM black-box attacks. (a) Restoration of the attacked forecast curve under the DAE defense when γ=−1. (b) Restoration of the attacked forecast curve under the DAE defense when γ=1.

Fig 12 illustrates the forecasting results under the DAE defense against DC-MI-FGSM black-box attacks from different directions. As shown in the figure, adversarial perturbations significantly distort the original forecasting curves, causing pronounced deviations from the true wind power trajectories. After applying the DAE-based defense, the attacked forecasts are effectively restored and closely follow the ground-truth curves, while preserving the original temporal patterns and dynamic trends. This indicates that the proposed DAE can not only reduce forecast errors but also maintain temporal consistency. Moreover, the results demonstrate that the DAE trained under the white-box setting generalizes well to black-box attack scenarios, providing strong visual evidence of its robustness and defensive effectiveness.

By analyzing and comparing the experimental results in the black-box environment, it can be concluded that the DAE defense algorithm trained in the white-box environment can resist the DC-MI-FGSM black-box attacks, effectively restoring the forecast curves. Compared with AT, DAE achieves a more significant reduction in the forecast errors caused by DC-MI-FGSM black-box attacks. Additionally, AT fails to defend against the DC-MI-FGSM black-box attacks at lower perturbation strengths, whereas DAE remains effective. These results highlight the generalization capability of DAE in the black-box environment.

## Conclusions

In this paper, we investigate adversarial attacks and defenses in wind power forecasting. To improve attack stealthiness, we propose the DC-MI-FGSM attack method. DC-MI-FGSM incorporates a momentum-based optimization mechanism during the iterative process while constraining the perturbation dimensions of input samples, enabling the generation of adversarial examples that are both highly effective and difficult to detect. To counteract such attack, we further develop a DAE-based preprocessing strategy. By leveraging a denoising defense mechanism, the DAE maps adversarial samples back to their corresponding clean representations, thereby significantly enhancing the robustness of the forecasting model.

The performance of the proposed attack and defense methods is systematically evaluated under white-box and black-box scenarios. For the attack, DC-MI-FGSM successfully drives the forecast curves to deviate upward or downward from the original trajectories while preserving the underlying temporal dynamics. Compared with existing attack methods, DC-MI-FGSM introduces smaller perturbations to the input samples, resulting in superior stealthiness. Meanwhile, it achieves stronger degradation of forecasting accuracy under both white-box and black-box conditions. For the defense, the DAE effectively mitigates the impact of DC-MI-FGSM attacks and significantly restores the corrupted forecast curves. Moreover, the DAE model trained in the white-box environment exhibits strong defensive performance against black-box attacks, demonstrating excellent generalization capability. In addition, the proposed DAE-based defense consistently outperforms adversarial training in reducing forecast errors under adversarial perturbations, while having a negligible impact on the original forecasting accuracy.

This study primarily focuses on the stealthiness of adversarial attacks and preprocessing-based defense strategies in wind power forecasting. Despite the encouraging results, several limitations remain and indicate promising directions for future research. First, this work concentrates on preprocessing-based defenses and does not investigate hybrid defense strategies. For example, integrating DAE with adversarial training to further enhance robustness remains an important topic for future exploration. Second, adversarial attack detection is not addressed in this study. Developing reliable detection mechanisms capable of identifying stealthy adversarial perturbations under complex operating conditions remains an open research challenge. Third, although the proposed methods demonstrate strong effectiveness in experimental evaluations, practical issues related to real-time deployment in operational wind power forecasting systems have not been examined and warrant further investigation.

## Supporting information

S1 Data Raw data. (CSV)
